# Opium, tobacco, and alcohol use in relation to oesophageal squamous cell carcinoma in a high-risk area of Iran

**DOI:** 10.1038/sj.bjc.6604369

**Published:** 2008-05-13

**Authors:** D Nasrollahzadeh, F Kamangar, K Aghcheli, M Sotoudeh, F Islami, C C Abnet, R Shakeri, A Pourshams, H A Marjani, M Nouraie, M Khatibian, S Semnani, W Ye, P Boffetta, S M Dawsey, R Malekzadeh

**Affiliations:** 1 Digestive Disease Research Center, Medical Sciences/University of Tehran, Tehran, Iran; 2 Medical Epidemiology and Biostatistics, Karolinska Institute, Stockholm, Sweden; 3 Division of Cancer Epidemiology and Genetics, National Cancer Institute, Bethesda, MD, USA; 4 International Agency for Research on Cancer, Lyon, France; 5 Golestan Research Center of Gastroenterology and Hepatology, Gorgan University of Medical Sciences, Gorgan, Iran

**Keywords:** oesophageal cancer, Iran, opium, tobacco, alcohol

## Abstract

The very high incidence of oesophageal squamous cell carcinoma (ESCC) in Golestan Province in northeastern Iran was suggested by studies in the 1970s as partly due to opium use, which is not uncommon in this area, but based on limited numbers. From December 2003 to June 2007, we administered a validated structured questionnaire to 300 ESCC cases and 571 controls, matched on neighbourhood of residence, age (±2 years), and sex. We used conditional logistic regression models to calculate odds ratios (ORs) and 95% confidence intervals (95% CIs) adjusted for potential confounders. Compared with those who used neither tobacco nor opium, risk of ESCC was increased in those who used tobacco only (OR, 95% CI: 1.70, 1.05–2.73), in those who used opium only (2.12, 1.21–3.74), and in those who used both tobacco and opium (2.35, 1.50–3.67). All forms of tobacco use (cigarettes, hookah, and nass) were associated with higher ESCC risk. Similarly, use of both crude opium and other forms of opium were associated with higher risk. Alcohol consumption was seen in only 2% of the cases and 2% of the controls, and was not associated with ESCC risk.

Incidence and mortality rates of oesophageal squamous cell carcinoma (ESCC) in the eastern part of Golestan Province, northeast Iran, are among the highest in the world (Mahboubi *et al*, 1973; Kamangar *et al*, 2007). Studies conducted by the International Agency for Research on Cancer (IARC) and Institute of Public Health Research (IPHR) of the University of Tehran in the 1970s suggested that opium consumption was one causal factor (Cook-Mozaffari *et al*, 1979). Two studies found an almost six-fold higher prevalence of appreciable levels (⩾1 *μ*g ml^−1^) of urinary morphine among residents of the high-risk areas than in lower risk areas (Joint Iran–IARC Study Group, 1977; Ghadirian *et al*, 1985). One of these studies prematurely terminated in 1979, found a two-fold increased positivity rate for urinary opium metabolites among spouses of 41 case subjects compared with matched controls (Ghadirian *et al*, 1985). Opium use is still prevalent in Golestan Province, especially among rural men (Pourshams *et al*, 2004), and may contribute to the high rates of ESCC in this area.

Heavy alcohol consumption and cigarette smoking are major causes of ESCC in most parts of the world (Tuyns, 1983; Brown *et al*, 1994, 2001), whereas in the 1970s alcohol consumption was rare in Golestan Province, especially among rural people (Cook-Mozaffari *et al*, 1979). These same studies showed that cigarette smoking, although associated with a two-fold increased risk of ESCC in Golestan (Cook-Mozaffari *et al*, 1979), was lower than in western countries, where it increases risk by 3–5-fold. Golestan people may smoke tobacco using a hookah (water pipe) or chew tobacco as nass, a combination of tobacco, ash, and lime (Joint Iran–IARC Study Group, 1977; Cook-Mozaffari *et al*, 1979; Pourshams *et al*, 2004), but these have been little studied in relation to ESCC.

In 2003, we initiated a new case–control study to assess the role of various risk factors in ESCC in Golestan Province.

## MATERIALS AND METHODS

### Case and control selection

The study area included the counties of Gonbad, Minoodasht, Kalaleh, Azadshahr, and Ramian in eastern Golestan province. Approximately half of the residents of the study area are of Turkmen ethnicity, and the rest Persians, Kurds, Turks, and others. Cases were recruited at Atrak Clinic in Gonbad City, the largest in eastern Golestan Province, and is the only specialised clinic covering ESCC. Local physicians were asked to refer any suspected upper gastrointestinal tract cancers to Atrak Clinic. A local cancer registry showed that approximately 70% of the incident oesophageal cancer cases during the study period were referred to Atrak Clinic (unpublished).

Case subjects were recruited from December 2003 to June 2007, had histopathologically confirmed ESCC, and agreed to participate in the study. Other eligibility criteria were being at least 18 years of age, residing in the study area at the time of registration, and having no history of concurrent cancer in other organs or history of previous cancer in any organ. All the patients underwent oesophago-gastro-duodenal videoendoscopy at Atrak Clinic, and biopsy samples were sent to the Tehran University Digestive Disease Research Center (DDRC), in Tehran, where they were examined by experienced pathologists.

For each case subject, we attempted to select two population-based control subjects matched to the case for neighbourhood of residence or village, age (±2 years), and sex. However, for a minority of cases (∼10%), we found only one matched control. In both rural villages and urban areas, the family health census, which is conducted annually by the Iranian Public Health Network, was used to identify eligible controls. In each village, our interview group identified all of the potentially eligible controls and randomly selected one to interview. If the first person could not be interviewed for any reason, the second person on the list was invited, and so forth. In urban areas, a list of the eligible controls was ordered by geographic proximity to the case's residence, with the list starting from the eligible person living closest to the case's residence. If the first person was not available for interview, then the second closest person was invited, and so forth.

### Data collection

The conduct of this study was reviewed and approved by the Institutional Review Boards of the DDRC, the US National Cancer Institute (NCI), and IARC. After obtaining written informed consent from each subject, two trained interviewers, a nurse and a physician, administered a structured general questionnaire. Also, a nutritionist administered a validated semiquantitative food frequency questionnaire that was specifically developed for this area (Malekshah *et al*, 2006). During the pilot phase, which extended from March 2002 to November 2003 (Islami *et al*, 2004), interviewers were given a thorough training, questionnaires were tested for reliability and validity, and databases and other facilities were improved. To diminish interindividual variation, a limited number of staff conducted the face-to-face interviews and no proxies were used. Case subjects were interviewed on the day of diagnostic upper gastrointestinal endoscopy at the Atrak Clinic. Rural controls were interviewed in village health houses, urban controls in urban health centres except those selected from Gonbad City, who were interviewed at a research facility established by DDRC for an ongoing cohort study on upper gastrointestinal tract cancers (Pourshams *et al*, 2004).

The questionnaire covered lifelong history of tobacco use, including smoking cigarettes, using hookah (water pipe), and chewing nass, with starting and stopping ages and daily amount used. Also, any change in amount and type of tobacco use was recorded. Ever-cigarette smokers, ever-hookah users, and ever-nass users were those who had smoked, used, or chewed cigarettes, hookah, or nass (respectively) at least weekly for a period of 6 months or more. Ever-tobacco users were individuals who had done any of the above, further classified as current and former users. Current users were those who had practised any of these habits within 1 year before the interview. Former users were those who had stopped their habit at least 1 year prior to the interview.

The general questionnaire covered opium use, which in Golestan includes teriak (crude opium), shireh (a refined opium extract) (Hewer *et al*, 1978), sukhteh (opium dross left in pipes after smoking opium), and heroin. Teriak and shireh can be smoked or ingested, sukhteh is usually ingested, and heroin is usually injected. Opium users, defined as subjects who had consumed opium at least once per week for a minimum of 6 months, were asked about type(s) of opiate used, the route of administration, the age they started and stopped, and the amount consumed. Study participants were also asked whether they had been given opiates during infancy or childhood.

Alcohol consumption in the study area is uncommon (Pourshams *et al*, 2004). Subjects were asked about type of alcohol used (beer, imported spirit, country spirit, and others), starting and stopping ages, and the amount and frequency of use.

Detailed information was also collected on age, sex, education, ethnicity, place of residence, and other potential confounders. Responses to questions regarding opium, tobacco, and alcohol consumption in this area were previously validated (Abnet *et al*, 2004; Pourshams *et al*, 2004).

### Statistical analysis

For each exposure (opium, cigarette, hookah, nass, and alcohol), average intensity, total duration, and cumulative use (average intensity multiplied by duration of use) were categorised into three groups: no use, low use (⩽median use in controls), and high use (>median use). Age of first use was similarly categorised. Percentages were tabulated for each of these categories by case status. Conditional logistic regression models were used to calculate unadjusted and adjusted odds ratios (ORs) and 95% confidence intervals (95% CIs). In all analyses, nonusers were the referent group. By design, case and control subjects were matched for age, sex, and place of residence. Results were adjusted for education (as a marker of socioeconomic status) and ethnicity (Turkmen *vs* non-Turkmen). *P*-values for trend were obtained from adjusted models by assigning values of 0, 1, and 2 to no use, low use, and high use, respectively.

We also analysed and present ORs (95% CIs) for different types of tobacco use and opium use, mutually adjusted for each other, and for education, ethnicity, and total intake of fruit and vegetables. Population attributable risks were obtained from these mutually adjusted models, using the prevalence of tobacco and opium use in the control subjects. Two-sided *P*-values ⩽0.05 were considered as statistically significant. All statistical analyses were done using Stata Software, version 10.0 (StataCorp., College Station, TX, USA).

## RESULTS

Demographic characteristics of the study participants are summarised in Table 1. A total of 300 ESCC case subjects were enrolled in this study. Of these, 271 had two matched controls and 29 had one in a total of 571. Mean age (s.d.) was 64.5 (10.1) for cases and 64.3 (10.4) for controls. Of the cases, 50% were male, and 27% lived in urban areas. Matched for age, sex, and place of residence, 57% of cases and 55% of controls were of Turkmen ethnicity.

In all, 90 cases (30%) and 106 controls (18%) had ever used opium (Table 2). Opium use was common in both men and in women; 32% of men (42% of cases and 27% of controls) and 13% of women (18% of cases and 11% of controls) had used opium. Prevalence of ever use increased with age, but was common in all age groups. Among controls, 11% of those aged ⩽50 years, 19% of those 51–70 years, and 21% of those >70 years had ever used opium. Ever use was associated with increased ESCC risk, with an adjusted OR (95% CI) of 2.00 (1.39–2.88), and for use earlier than a year prior to diagnosis, was 1.92 (1.30–2.84). Average intensity of use, duration of use, cumulative use, and age at first use all showed significant dose–response associations with ESCC risk (all *P*-values for trend <0.001). No subject was a sukhteh or heroin user. Using the teriak or shireh form of opium was associated with higher risk of ESCC; adjusted ORs (95% CIs) for teriak use only, shireh use only, or use of both were 1.62 (1.09–2.40), 3.41 (1.35–8.60), and 8.80 (2.28–33.9), respectively. Both routes of administration also increased risk of ESCC; adjusted ORs (95% CIs) for smoking only, ingestion only, and both were 1.67 (1.06–2.63), 1.90 (1.09–3.32), and 6.61 (2.42–18.0), respectively. Of the 250 cases and 509 controls who knew whether they were given opium in their childhood, 23 cases (9%) and 37 controls (7%) stated that they had received opiates during that time; the adjusted OR (95% CI) was 1.11 (0.61–2.03).

A total of 67 cases (22%) and 99 controls (17%) ever regularly smoked cigarettes, and this habit was associated with a nearly significant increased ESCC risk, adjusted OR (95% CI) being 1.47 (0.98–2.21). Risk was higher in current than in former smokers, in those who smoked above the median intensity among smoking controls (11 cigarettes per day), for longer than the median duration among smoking controls (21 years), in those whose cumulative pack-years was higher than median among smoking controls (13.5 pack-years), and in those who started smoking earlier than the median age of starting among controls (25 years); *P*-values for trend were significant for all of these comparisons (Table 3).

Of cases, 20 (7%) and 23 controls (4%) had ever used hookah (Table 3). Ever regularly smoking a hookah was associated with a nearly significant increased risk of ESCC, with an adjusted OR (95% CI) of 1.85 (0.95–3.58). Higher average intensity of use (*P* for trend=0.03) and younger age of starting (*P* for trend=0.05), but not duration of use (*P* for trend=0.21) or cumulative use (*P* for trend=0.11), showed significant associations with ESCC risk. Of cases, 44 (15%) and 48 controls (8%) had ever chewed nass. Ever regularly chewing nass showed increased ESCC risk, with an adjusted OR (95% CI) of 1.99 (1.21–3.28). Average intensity of use (*P* for trend=0.004), duration of use (*P* for trend=0.02), cumulative use (*P* for trend=0.007), and age of first use (*P* for trend=0.01), all showed significant associations with risk (Table 3).

Adjusted ORs (95% CIs) for cigarette use alone, hookah use alone, nass chewing alone, and combinations of two or more types were 1.50 (0.92–2.43), 1.69 (0.76–3.77), 2.91 (1.46–5.77), and 2.11 (1.15–3.86), respectively (Table 4). Compared with using neither (Table 4), tobacco use alone, opium use alone, and a combination of the two were all significantly associated with higher ESCC risk, with adjusted ORs (95% CIs) of 1.70 (1.05–2.73), 2.12 (1.21–3.74), and 2.35 (1.50–3.67), respectively. Using results from Table 4, tobacco and opium use accounted for 30% of the cases in this study.

Only 21 subjects, 7 cases (2%) and 14 controls (2%), had ever used alcohol for 6 months or more, adjusted OR (95% CI) 0.93 (0.37–2.34). There was no significant association between intensity, duration, cumulative use, or age of first use of alcohol with ESCC risk (data not shown).

## DISCUSSION

Our study showed that opium consumption, tobacco smoking, and chewing, but not alcohol consumption, are associated with higher risk of ESCC in Golestan Province. The area is well-placed to study the relationship between opium use and ESCC, both of which are common. A large proportion of the world's opium is produced in Afghanistan, which borders Iran, and it is still commonly used in this region as a traditional treatment of pain, diarrhoea, and insomnia. An ecologic study in northern Iran found that 50% (20 out of 40) of adults in ESCC high-risk areas, compared with only 11% (10 out of 90) of those in medium- or low-risk areas, tested positive for urine morphine metabolites (Joint Iran–IARC Study Group, 1977). In a subsequent study of 1590 rural individuals, the prevalence of appreciable levels (⩾1 *μ*g ml^−1^) of urinary morphine metabolites was almost six-fold higher among residents of high-risk *vs* low-risk areas (Ghadirian *et al*, 1985). Among the spouses of 41 cases and 41 age-matched controls, there was a two-fold, nonsignificant, increased risk associated with the presence of urinary morphine metabolites.

Previous studies in Iran (Cook-Mozaffari *et al*, 1979; Ghadirian *et al*, 1985) avoided using questionnaires to ask about opium use, partly because it can be a sensitive subject in many populations, which may limit the validity of questionnaire responses. This was tested during the pilot phase of an ongoing cohort study in Golestan by interviewing subjects twice and collecting urine samples twice, 2 months apart (Abnet *et al*, 2004). Overall agreement between the responses in the two interviews for ever using opium was excellent and above 0.99, the corresponding *κ*-statistic being 0.96 (Abnet *et al*, 2004). The validity of self-reported current opium use was also high. Using the presence of codeine or morphine in the urine, self-report had a sensitivity of 0.93 and specificity of 0.89 (Abnet *et al*, 2004), so we accepted questionnaire to document opium use. The results of our study showed a two-fold increased risk of ESCC associated with opium use. People in Golestan may start using opiates to alleviate pain prior to their cancer diagnosis and, therefore, reverse causality is a concern. However, excluding the cases and controls who had recently started using opium from the analysis made no material difference in the study results, and younger age at first use was a strong predictor of cancer risk. Limiting the analysis to tobacco nonusers, the results remained essentially unchanged. The percentage of opium users in ESCC case subjects (30%) found in this study is close to the percentage (33%) found in the pilot phase study (Islami *et al*, 2004).

The two forms of opium used by the subjects of this study were teriak (crude opium) and shireh (refined opium). Shireh is usually made by boiling teriak (or a mixture of teriak and opium pyrolysates obtained after smoking opium) with water, filtering the mixture several times, and, then, evaporating the filtrate until a gummy consistency is achieved. Both teriak and shireh can be smoked or ingested. We found that consumption of both teriak and shireh are common, and both are associated with increased risk of ESCC, also both, smoking and ingesting, alone or in combination.

Neither teriak nor shireh themselves were mutagenic in the Ames test (Hewer *et al*, 1978). However, either smoking or inhaling the smoke involves exposure to potential carcinogens, including polycyclic aromatic hydrocarbons (PAHs) to which industrial exposure may cause cancers of the lung, bladder, and skin (Denissenko *et al*, 1996; Boffetta *et al*, 1997; Hecht, 2003). Smoke condensates from opium and morphine cause mutations in *Salmonella typhimurium* (Hewer *et al*, 1978; Malaveille *et al*, 1982), sister chromatid exchanges in human lymphocytes (Perry *et al*, 1983), and morphological transformations in cultured Syrian hamster embryo cells (Friesen *et al*, 1985), whereas crude opium itself is not a mutagen (Hewer *et al*, 1978; Malaveille *et al*, 1982). The mechanisms responsible for the carcinogenic effects of opium and shireh need investigation.

In western countries, cigarette smoking increases ESCC risk by approximately 3–5-fold (Tuyns, 1983; Brown *et al*, 1994, 2001). However, in some high-risk areas, such as Linxian (China), smoking plays a much less significant role in the aetiology of ESCC, and the relative risk for ever-cigarette smoking is only 1.3 (Tran *et al*, 2005). The case–control study conducted by IARC and IPHR in the 1970s in Golestan Province showed an almost two-fold risk of ESCC associated with tobacco smoking (Cook-Mozaffari *et al*, 1979) and adjusted OR of 1.47 confirms a weak-to-moderate association in Golestan. Discordance between smoking prevalence and ESCC rates in women and men in these high risk areas, is in agreement with the finding of smoking is not a strong risk factor in these population. In Linxian, <1% of women and approximately 70% of men smoke (Abnet *et al*, 2001), yet ESCC rates are nearly equal in the two sexes as in Golestan, where the prevalence of ever smoking ranges from 1% in rural women to 39% in urban men (Pourshams *et al*, 2004), suggesting that most ESCC cases in Linxian and Golestan are due to other factors. This relatively weak association between smoking and ESCC risk may also be due in part to the relatively low cumulative tobacco exposure; median cumulative use among smoking controls in this study was only 13.5 pack-years.

The association of ESCC with smoking hookah or chewing nass has been little studied. In our study, although the CIs are wide, the point estimates suggest that these are at least as strong risk factors as cigarette smoking. Regular use of hookah may involve exposure to large amounts of tobacco combustion products. Because nass is a mixture of tobacco, ash, and lime (Joint Iran–IARC Study Group, 1977; Cook-Mozaffari *et al*, 1979; Pourshams *et al*, 2004), it exposes users to carcinogens both in unburned tobacco (e.g., nitrosamines, PAHs, and aldehydes) and in ash (e.g., PAHs). In our study, both the intensity of hookah use and the intensity and duration of nass use showed positive trends with ESCC risk, but not duration of hookah use, perhaps partly due to people changing from hookah to cigarettes. Consistent with our findings, snus (Scandinavian moist snuff) use is associated with a three-fold significant increased risk of ESCC (Zendehdel *et al*, 2008). However, there was no association between hookah or nass use and ESCC risk in the previous IARC–IPHR case–control study (Cook-Mozaffari *et al*, 1979).

Like tobacco smoking, alcohol use is a major cause of ESCC in Western countries (Jensen, 1979; Tuyns, 1983; Boffetta and Garfinkel, 1990; Brown *et al*, 1994, 2001), but not in Linxian (Tran *et al*, 2005), or Iran (Joint Iran–IARC Study Group, 1977; Pourshams *et al*, 2004), where consumption is very limited. In the West, alcohol intake is associated with a dose–response increase in ESCC risk, and heavy consumption increases risk by 5–15-fold (Tuyns, 1983; Boffetta and Garfinkel, 1990; Brown *et al*, 1994; Baan *et al*, 2007). In our study, approximately 2% of cases and 2% of controls had ever regularly used alcohol, which was not significantly associated with ESCC risk.

Overall agreement between responses to each of the cigarette, nass, and alcohol questions in the two interviews was above 0.99, and the corresponding *κ*-statistics were all above 0.95 (Abnet *et al*, 2004), showing excellent reliability.

Although the ESCC incidence rate is still high in Golestan Province, it has declined in the past few decades, particularly among younger people (Semnani *et al*, 2006). Factors contributing to this decline may include improved socioeconomic status, more widespread access to piped water, and more fresh fruits and vegetable use due to greater availability of refrigerators. Also, the prevalence of opium use in our study was approximately half of that reported in the 1970s (Ghadirian *et al*, 1985), when sukhteh use was common (Hewer *et al*, 1978), but not reported by our subjects.

The strengths of this study include the relatively large sample size, strict matching for area of residence to avoid possibility of confounding by region, verification of the reliability and validity of questionnaires, histologic confirmation of all case diagnoses, and appropriate sensitivity analyses. Limitations include recall bias, but as most subjects were illiterate or with little education, and were unaware of the study hypotheses, it is unlikely that reported opium, tobacco, or alcohol use are differentially higher in case than in control subjects.

## Figures and Tables

**Table 1:** Demographic characteristics of ESCC cases and matched controls

	**ESCC cases**	**Matched controls**
	***N* (%)**	***N* (%)**
Total	300 (100)	571 (100)
		
*Age (years)*		
⩽50	32 (11)	61 (11)
51–60	81 (27)	144 (25)
61–70	86 (29)	177 (31)
>70	101 (34)	189 (33)
		
*Gender*		
Male	150 (50)	278 (49)
Female	150 (50)	293 (51)
		
*Place of residence*		
Urban	82 (27)	150 (26)
Rural	218 (73)	421 (74)
		
*Ethnicity*		
Turkmen	171 (57)	312 (55)
Non-Turkmen	129 (43)	259 (45)

ESCC=oesophageal squamous cell carcinoma.

Although cases and controls were individually matched, the percentages of cases and controls are not necessarily equal in each age, gender, or place of residence category, because some cases have one matched control and others have two matched controls.

**Table 2:** Opium use in ESCC cases and matched controls

	**ESCC cases**	**Matched controls**	**Unadjusted**	**Adjusted**	
	***N* (%)**	***N* (%)**	**OR (95% CI)**	**OR (95% CI)^a^**	***P* for trend^b^**
*Opium use*					
Never	210 (70)	465 (81)	Referent	Referent	—
Ever	90 (30)	106 (18)	1.95 (1.36–2.78)	2.00 (1.39–2.88)	
					
*Average intensity*					
Never used	210 (70)	465 (81)	Referent	Referent	<0.001
⩽ median (1.5 units per day)^c^^,^^d^	42 (14)	53 (9)	1.75 (1.11–2.75)	1.72 (1.08–2.72)	
> median	48 (16)	53 (9)	2.19 (1.37–3.51)	2.38 (1.47–3.85)	
					
*Duration*					
Never used	210 (70)	465 (81)	Referent	Referent	<0.001
⩽ median (8.5 years)^d^	34 (11)	53 (9)	1.44 (0.88–2.39)	1.47 (0.89–2.44)	
> median	56 (19)	53 (9)	2.37 (1.54–3.65)	2.47 (1.59–3.84)	
					
*Cumulative use* e					
Never used	210 (70)	465 (81)	Referent	Referent	<0.001
⩽ median (15.5 unit-years)^c^^,^^d^	41 (14)	53 (9)	1.74 (1.10–2.75)	1.73 (1.09–2.74)	
> median	49 (16)	53 (9)	2.19 (1.38–3.47)	2.34 (1.45–3.78)	
					
*Age started*					
Never used	210 (70)	465 (81)	Referent	Referent	<0.001
> median	29 (10)	50 (9)	1.26 (0.74–2.16)	1.26 (0.74–2.16)	
⩽ median (50 years)^d^	61 (20)	56 (10)	2.50 (1.63–3.84)	2.64 (1.71–4.09)	
					
*Type of opium used*					
Never used	210 (70)	465 (81)	Referent	Referent	—
Crude opium (teriak) only	66 (22)	95 (17)	1.56 (1.06–2.31)	1.62 (1.09–2.40)	
Refined opium (shireh) only	14 (5)	8 (1)	3.84 (1.57–9.42)	3.41 (1.35–8.60)	
Both	10 (3)	3 (1)	7.36 (1.99–27.2)	8.80 (2.28–33.9)	
					
*Route of administration*					
Never used	210 (70)	465 (81)	Referent	Referent	—
Smoked only	45 (15)	62 (11)	1.67 (1.06–2.59)	1.67 (1.06–2.63)	
Ingested only	30 (10)	38 (7)	1.86 (1.07–3.21)	1.90 (1.09–3.32)	
Both	15 (5)	6 (1)	5.41 (2.05–14.2)	6.61 (2.42–18.0)	

95% CI=95% confidence interval; ESCC=oesophageal squamous cell carcinoma; OR=odds ratio. ORs were obtained from conditional logistic regression models.

a Adjusted for education and ethnicity.

b *P* for trend was obtained from conditional logistic regression models by assigning values of 0, 1, and 2 to no use, below median, and above median use, respectively.

c Each unit is one ‘nokhod’, which is approximately equal to 0.2 g.

d We used the median in control subjects as the dividing cut point.

e Cumulative use was calculated by multiplying intensity of use (per day) by duration of use (in years).

**Table 3:** Tobacco use in ESCC cases and matched controls

	**ESCC cases**	**Matched controls**	**Unadjusted**	**Adjusted**	
	***N* (%)**	***N* (%)**	**OR (95% CI)**	**OR (95% CI)^a^**	***P* for trend^b^**
*Cigarette smoking* c					
Never	232 (78)	471 (83)	Referent	Referent	—
Ever	67 (22)	99 (17)	1.35 (0.92–2.00)	1.47 (0.98–2.21)	
					
*Cigarette smoking*					
Never used	232 (78)	471 (83)	Referent	Referent	0.05
Former smoker	34 (11)	56 (10)	1.24 (0.75–2.03)	1.34 (0.80–2.23)	
Current smoker	33 (11)	43 (8)	1.49 (0.90–2.48)	1.63 (0.97–2.76)	
					
*Average intensity*					
Never used	232 (78)	471 (83)	Referent	Referent	0.01
⩽ median (11 cigarettes per day)^d^	22 (7)	49 (9)	0.89 (0.51–1.56)	0.98 (0.55–1.75)	
> median	45 (15)	50 (9)	1.84 (1.13–2.99)	1.98 (1.20–3.25)	
					
*Duration*					
Never used	232 (78)	471 (83)	Referent	Referent	0.04
⩽ median (21 years)^d^	30 (10)	50 (9)	1.18 (0.72–1.94)	1.27 (0.76–2.11)	
> median	37 (12)	49 (9)	1.58 (0.95–2.62)	1.73 (1.03–2.93)	
					
*Cumulative use* e					
Never used	232 (78)	471 (83)	Referent	Referent	0.05
⩽ median (13.5 pack-years)^d^	31 (10)	50 (9)	1.22 (0.74–2.01)	1.36 (0.81–2.27)	
> median	36 (12)	49 (9)	1.52 (0.91–2.53)	1.61 (0.95–2.74)	
					
*Age started*					
Never used	232 (78)	471 (83)	Referent	Referent	0.02
> median	23 (8)	46 (8)	0.98 (0.57–1.68)	1.07 (0.62–1.86)	
⩽ median (25 years)^d^	44 (15)	53 (9)	1.75 (1.08–2.84)	1.90 (1.15–3.11)	
					
*Hookah smoking*					
Never	280 (93)	548 (96)	Referent	Referent	—
Ever	20 (7)	23 (4)	1.81 (0.95–3.43)	1.85 (0.95–3.58)	
					
*Average intensity*					
Never used	280 (93)	548 (96)	Referent	Referent	0.03
⩽ median (3 times per day)^d^	11 (4)	18 (3)	1.29 (0.59–2.84)	1.31 (0.58–2.96)	
> median	9 (3)	5 (1)	3.51 (1.17–10.5)	3.57 (1.17–11.0)	
					
*Duration*					
Never used	280 (93)	548 (96)	Referent	Referent	0.21
⩽ median (19 years)^d^	15 (5)	12 (2)	2.52 (1.14–5.55)	2.45 (1.09–5.49)	
> median	5 (2)	11 (2)	1.00 (0.34–2.90)	1.11 (0.38–3.30)	
					
*Cumulative use* e					
Never used	280 (93)	548 (96)	Referent	Referent	0.11
⩽ median (32 hookah-years)^d^	12 (4)	12 (2)	2.07 (0.87–4.88)	2.04 (0.84–4.99)	
> median	8 (3)	11 (2)	1.55 (0.62–3.87)	1.66 (0.65–4.22)	
					
*Age started*					
Never used	280 (93)	548 (96)	Referent	Referent	0.05
> median	7 (2)	11 (2)	1.55 (0.62–3.87)	1.38 (0.51–3.69)	
⩽ median (40 years)^d^	13 (4)	12 (2)	2.22 (0.99–5.00)	2.31 (0.98–5.48)	
					
*Nass chewing*					
Never	256 (85)	523 (92)	Referent	Referent	—
Ever	44 (15)	48 (8)	2.09 (1.28–3.42)	1.99 (1.21–3.28)	
					
*Average intensity*					
Never used	256 (85)	523 (92)	Referent	Referent	0.004
⩽ median (5.5 times per day)^d^	17 (6)	24 (4)	1.59 (0.78–3.25)	1.50 (0.73–3.09)	
> median	27 (9)	24 (4)	2.52 (1.38–4.61)	2.42 (1.31–4.47)	
					
*Duration*					
Never used	256 (85)	523 (92)	Referent	Referent	0.02
⩽ median (26.5 years)^d^	26 (9)	24 (4)	2.44 (1.32–4.49)	2.24 (1.21–4.16)	
> median	18 (6)	24 (4)	1.69 (0.83–3.41)	1.69 (0.83–3.44)	
					
*Cumulative use* e					
Never used	256 (85)	523 (92)	Referent	Referent	0.007
⩽ median (150 nass-years)^d^	22 (7)	25 (4)	2.19 (1.08–3.73)	1.81 (0.97–3.40)	
> median	22 (7)	23 (4)	2.20 (1.15–4.21)	2.21 (1.15–4.27)	
*Age started*					
Never used	256 (85)	523 (92)	Referent	Referent	0.01
> median	25 (8)	27 (5)	2.07 (1.06–4.04)	1.91 (0.98–3.75)	
⩽ median (40 years)^d^	19 (6)	21 (4)	2.11 (1.13–3.94)	2.06 (1.09–3.89)	

95% CI=95% confidence interval; ESCC=oesophageal squamous cell carcinoma; OR=odds ratio. ORs were obtained from conditional logistic regression models.

b Adjusted for education and ethnicity.

a *P* for trend was obtained from conditional logistic regression models by assigning values of 0, 1, and 2 to no use, below median, and above median use, respectively.

c Cigarette smoking status was unknown for one case and one control. Therefore, the total number of subjects in the cigarette smoking analysis is 299 cases and 570 controls.

d We used the median in control subjects as the dividing cut point.

e Cumulative use was calculated by multiplying intensity of use (per day) by duration of use (in years).

**Table 4:** The association between tobacco types and between tobacco and opium use, alone or in combination, and ESCC

	**ESCC cases**	**Matched controls**	**Unadjusted**	**Adjusted**
	***N* (%)**	***N* (%)**	**OR (95% CI)**	**OR (95% CI)^a^**
*Tobacco type used*				
Never used	196 (66)	432 (76)	Referent	Referent
Cigarettes only	41 (14)	68 (12)	1.32 (0.83–2.11)	1.50 (0.92–2.43)
Hookah only	12 (4)	18 (3)	1.66 (0.75–3.67)	1.69 (0.76–3.77)
Nass only	23 (8)	20 (4)	3.03 (1.53–5.96)	2.91 (1.46–5.77)
More than one type	27 (9)	32 (6)	2.12 (1.18–3.83)	2.11 (1.15–3.86)
				
Used neither tobacco nor opium	166 (56)	398 (70)	Referent	Referent
Used tobacco but not opium	43 (14)	66 (12)	1.68 (1.05–2.68)	1.70 (1.05–2.73)
Used opium but not tobacco	30 (10)	34 (6)	2.22 (1.27–3.87)	2.12 (1.21–3.74)
Used both tobacco and opium	60 (20)	72 (13)	2.20 (1.42–3.40)	2.35 (1.50–3.67)

95% CI=95% confidence interval; ESCC=oesophageal squamous cell carcinoma; OR=odds ratio. ORs were obtained from conditional logistic regression models.

a Adjusted for education, ethnicity, and total intake of fruit and vegetables.
